# Skin lesions by *Scedosporium apiospermum* and *Nocardia* pulmonary infection in an oncologic patient: a case report

**DOI:** 10.1186/s12879-023-08484-6

**Published:** 2023-08-09

**Authors:** M. Gavalda, A. Lorenzo, H. Vilchez, S. Gimenez, C. Calvo, L. Martin, M. Riera

**Affiliations:** 1 Internal Medicine, Hospital Universitari Son Espases, Palma, Spain; 2 Infectious Diseases Unit, Internal Medicine Department, Hospital Universitari Son Espases, Palma, Spain; 3 Oncology. Hospital Universitari Son Espases, Palma, Spain; 4 Pathology Department, Hospital Universitari Son Espases, Palma, Spain; 5 Fundació Institut d’Investigació Sanitària Illes Balears (IdISBa), Palma de Mallorca, 07120 Spain; 6 Hospital Universitari Son Espases, Valldemossa Road 79, Palma de Mallorca, Spain

**Keywords:** Fungal infection, *Scedosporium apiospermum*, *Nocardia*, Immunocompromised host, Voriconazole, Case report

## Abstract

**Background:**

Fungal infections, other than candidiasis and aspergillosis, are an uncommon entity. Despite this, emerging pathogens are a growing threat. In the following case report, we present the case of an immunocompromised patient suffering from two serious opportunistic infections in the same episode: the first of these, *Nocardia* multilobar pneumonia; and the second, skin infection by *Scedosporium apiospermum*. These required prolonged antibacterial and antifungal treatment.

**Case presentation:**

This case is a 71-year-old oncological patient admitted for recurrent pneumonias that was diagnosed for *Nocardia* pulmonary infection. Nervous system involvement was discarded and cotrimoxazole was started. Haemorrhagic skin ulcers in the lower limbs appeared after two weeks of hospital admission. We collected samples which were positive for *Scedosporium apiospermum* and we added voriconazole to the treatment. As a local complication, the patient presented a deep bruise that needed debridement. We completed 4 weeks of intravenous treatment with slow improvement and continued with oral treatment until the disappearance of the lesions occurs.

**Conclusions:**

Opportunistic infections are a rising entity as the number of immunocompromised patients is growing due to more use of immunosuppressive therapies and transplants. Clinicians must have a high suspicion to diagnose and treat them. A fluid collaboration with Microbiology is necessary as antimicrobial resistance is frequent.

## Background

Patients with neoplastic diseases are frequently at high risk of developing opportunistic infections. This susceptibility can be attributed to factors such as cancer or treatment-induced neutropenia, cellular immune defects, or local conditions like bronchial tree or biliary tract obstruction [1]. In this case, our patient experienced two opportunistic infections, which we will now present.

*Nocardia* is a type of aerobic gram-positive rod-shaped bacterium belonging to the *Actinomycetes* genus, primarily found in soil. Human infection is commonly caused by inhalation of Nocardia into the lungs. Although it primarily affects the lungs, it can also impact the brain, skin, eyes, or spread throughout the body. *Nocardia* infections are typically seen in individuals with compromised immune systems or chronic lung disease [2]. Symptoms often manifest as subacute pneumonia, characterized by dyspnoea, cough, and purulent expectoration. Fever is usually absent. Radiologically, common findings include consolidation, nodules, and cavitation. The diagnosis of *Nocardia* infection relies on culture-based techniques, typically utilizing bronchoalveolar lavage fluid or sputum samples. Subsequently, molecular techniques are employed for species identification. *Nocardia* infections are usually susceptible to treatment with amikacin, linezolid, and cotrimoxazole [3].

*Scedosporium apiospermum* is a filamentous fungus present in the environment as they have been found in rural soils, polluted water and composts [4]. It usually causes infection in severely immunocompromised patients, although it may affect healthy individuals with penetrating trauma. It can either present as a colonization (in cystic fibrosis, external ear or respiratory fungal ball), local infection (usually in skin, soft tissues and bone infection) or disseminated infection, with predilection for skin, sinuses, lungs and central nervous system [4–6]. The diagnosis is reached by conventional culture, although some diagnostic molecular methods have been tested. There are no solid recommendations about treatment. In vitro and in vivo data show that *S. apiospermum* is resistant to amphotericin-B and flucytosine and demonstrates variable susceptibility to itraconazole, voriconazole, posaconazole and micafungin. Currently, with the available data, voriconazole represents the first-line treatment. Surgery removal of localised lesions improves the outcome. The resolution of the factors that produce immunosuppression is the key for the treatment success [7].

## Case presentation

We present the case of a 71-year-old man with rectal adenocarcinoma admitted for fever and dyspnoea in March of 2022. The patient did not have any allergies; he was a former smoker and had hypertension, dyslipidaemia, benign prostatic hyperplasia and chronic obstructive pulmonary disease. In 2019 he was diagnosed of rectal adenocarcinoma and he received treatment with capecitabine, radiotherapy and underwent a surgery (abdominoperineal resection). In September of 2020 he presented a pulmonary metastasis therefore he started FOLFOX (oxaliplatin in combination with 5-fluorouracil and leucovorin) and Bevacizumab for 9 cycles and afterwards capecitabine and Bevacizumab for 3 more cycles. Due to pulmonary progression, he started the second line with FOLFORI (folinic acid, fluorouracil and irinotecan) and Cetuximab for 4 cycles until February of 2022.

In March of 2022 he had already been admitted twice for fever and dyspnoea, with a radiological image compatible with pneumonia. In the first admission he received treatment with amoxicillin-clavulanic acid and azithromycin and in the second hospitalization he was treated with piperacillin-tazobactam and levofloxacin as health care associated pneumonia. Cultures were negative, so he completed empirical treatment showing progressive clinical and analytical improvement. On both occasions, he was discharged afebrile, hemodynamically stable and without oxygen therapy needed.

**Fig. 1:**
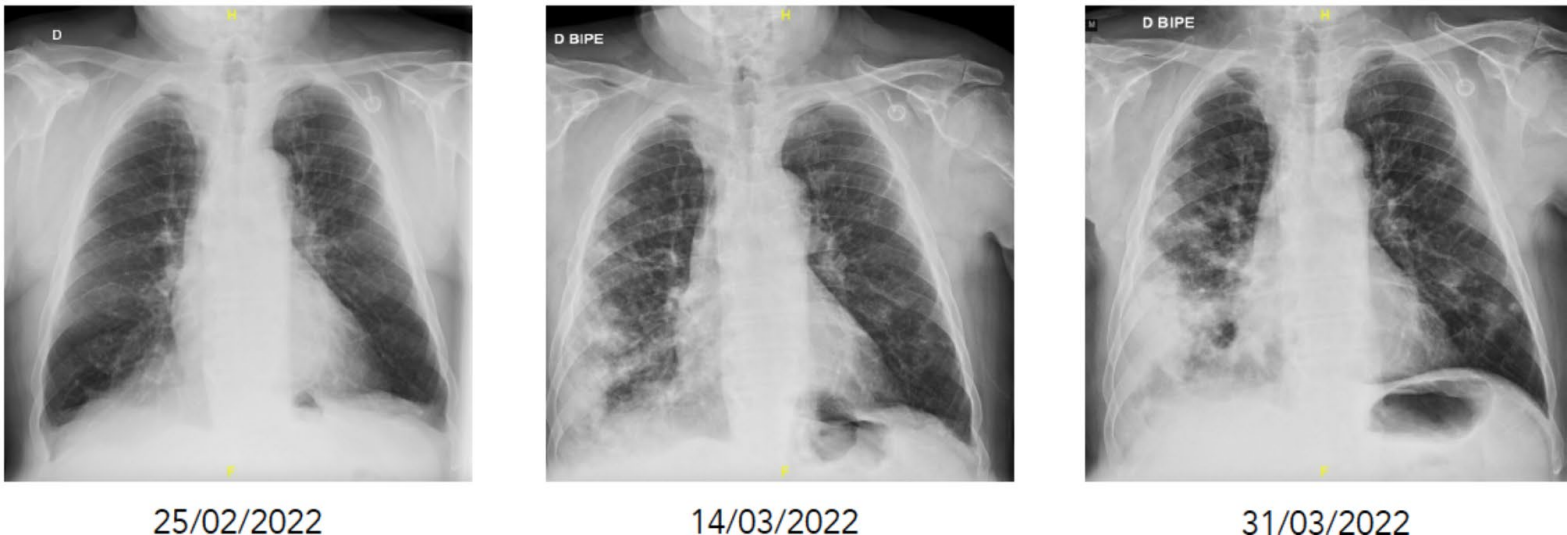
Radiological progression of Nocardia pneumonia

In the current episode, the 3rd admission in March of 2022, he is readmitted due to the reappearance of the symptoms a few days after being discharged. He presented elevated analytic acute phase reactants and radiographic worsening with multilobar involvement (Fig. 1). Empirical treatment was started with piperacillin-tazobactam 4 g/6 h and levofloxacin 500 mg/24 h. Also, a thoracic CT scan and a bronchoscopy were performed. The thoracic CT scan showed pulmonary lesions with necrosis and cavitation in both lungs, the metastasis had not changed in comparison with the previous scan. The bronchoscopy showed purulent secretions from both lungs, specially the right one. *Nocardia cyriacigeorgica* (> 100.000 CFU/ml) was successfully identified by MALDI-TOF in the samples of bronchial aspirate. Antibiotic treatment was changed to cotrimoxazole 800/120 mg/12 h, and brain infection was ruled out with a CT scan.

**Fig. 2:**
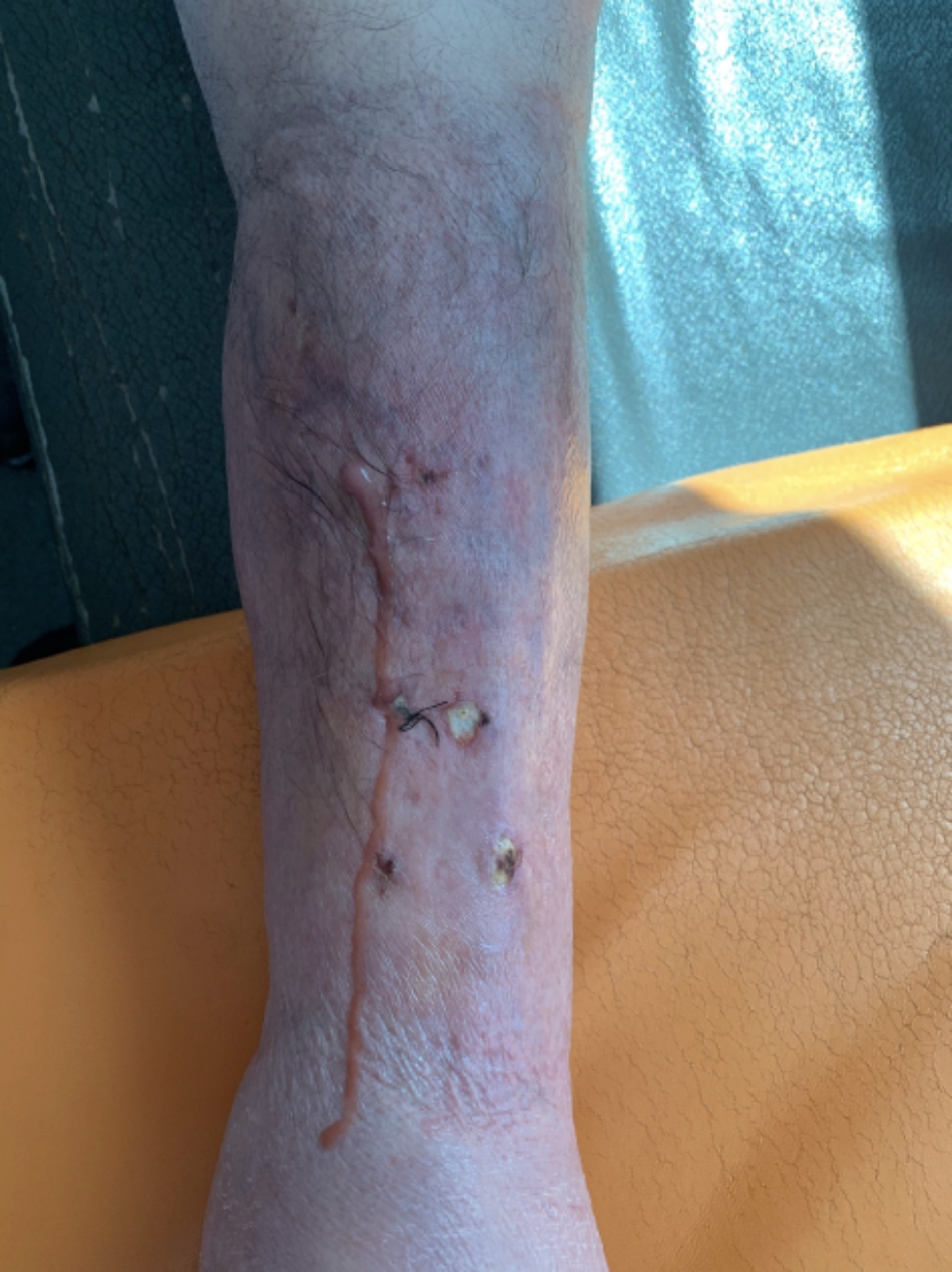
Haemorrhagic skin ulcers in the lower limbs

**Fig. 3:**
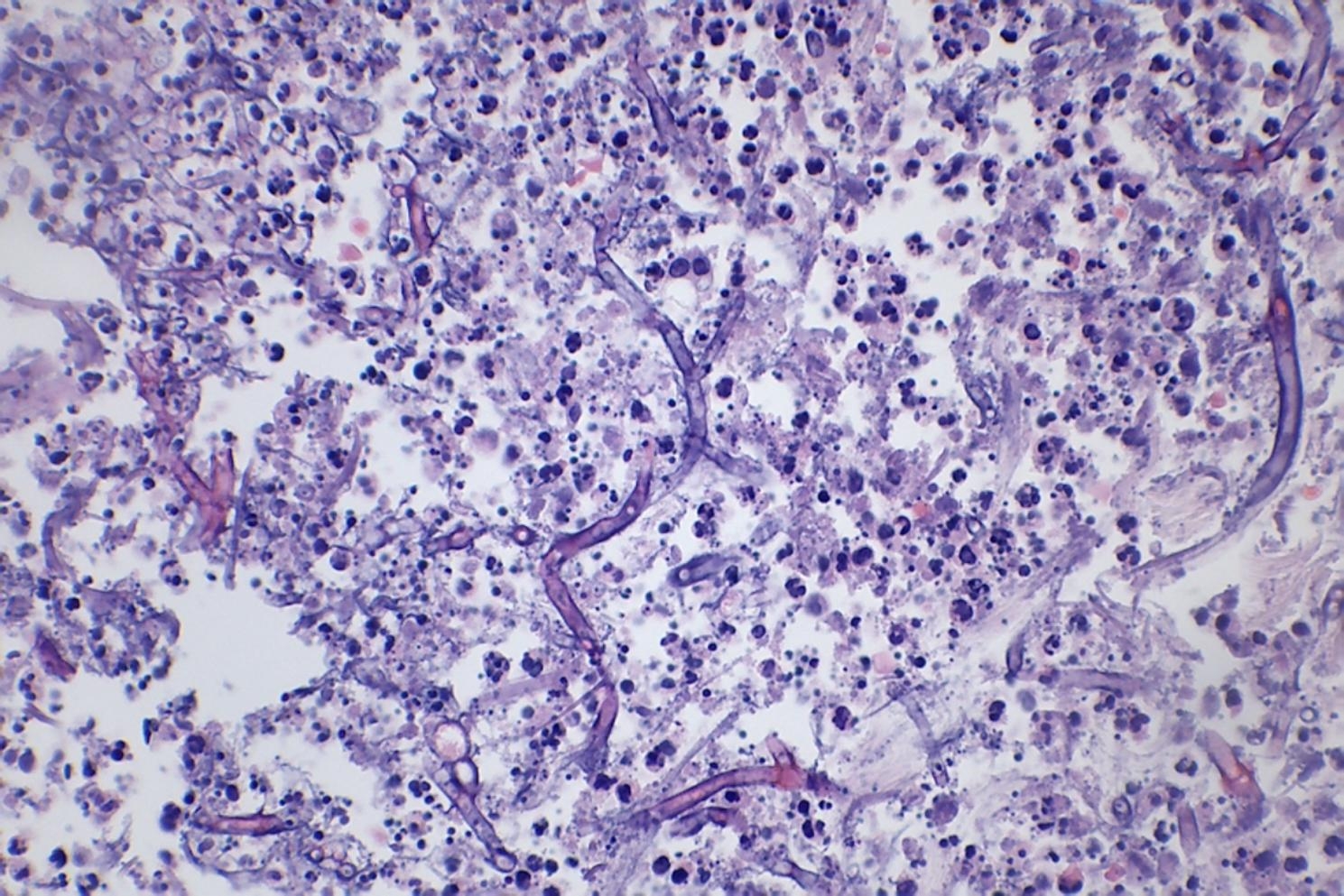
Abcessified areas and fungal structures with 90º ramification (pointed in arrows) as shown in Hematoxylin and Eosin

Two weeks later the patient presented haemorrhagic skin ulcers in the lower limbs, over an erythematous plaque (Fig. 2). The ulcers measured 5 mm of diameter and secreted a brown material. Both, a skin biopsy and a culture were performed. *Scedosporium apiospermum* was identified by calcofluor and lactophenol blue, as well as through MALDI-TOF technique in the samples from the skin biopsy. There were not any predisposing factors known as the patient had not had any trauma nor drowing incidents. The biopsy showed abcessified areas and fungal structures with 90º ramification with positivity for Hematoxylin-eosin, Grocott and Periodic acid-Schiff staining (Fig. 3). Voriconazole 300 mg/12 h was started after the results and was administered during 6 weeks at hospital admission with slow improvement. Deep infection was ruled out by lower limbs CT scan. As a local complication, the patient presented a deep bruise that needed debridement. The initial 4 weeks the patient received intravenous voriconazole and afterwards switched to oral treatment for the long term.

## Discusion and conclusions

Oncological patients are at a heightened risk of developing opportunistic infections, with approximately 80% of *Nocardia* infections occurring in immunocompromised individuals [2]. A review of 136 episodes of *Nocardia* infection at a cancer center revealed that 43.4% were associated with solid tumors, similar to our patient’s case, while the remainder were linked to haematological diseases [8]. Clinical differences exist between infections in immunocompromised and immunocompetent patients. Immunocompromised individuals are more likely to experience cavitation lesions in the lungs, disseminated infection, and eye involvement. Furthermore, mortality rates are significantly higher in this group, reaching 27% compared to 7% in immunocompetent patients [2].

Regarding identification, a multi-center study conducted in Spain found that the most commonly isolated species is *N. cyriacigeorgica*, as observed in our case [3]. Antimicrobial susceptibility varies among *Nocardia* species. In Spain, amikacin and linezolid exhibit activity against all species in a majority of cases (98.3% and 99.4% respectively), while carbapenems and clotrimazole are effective against most species (90.5% and 83.8% respectively) [9].

On the other hand, fungal infections are a growing entity globally. Emerging pathogens such as *Scedosporium* should not be ignored, as it was the second most frequently isolated filamentous fungus in a population-based survey in Spain [10]. Epidemiological studies in other countries also confirm this trend [11].

*Scedosporium* infection has been associated with corticosteroid use, chronic lung disease, diabetes and haematological malignancy and not usually with solid tumors [12–15] In the case of our patient, he had been receiving corticosteroid treatment while undergoing treatment for *Nocardia* pneumonia. *Scedosporium* has also been described in immunocompetent hosts after drowning in polluted wated or penetrating trauma [5, 6]. The most common forms of infection in immunocompetent hosts are keratitis, endophthalmitis, otitis, sinusitis, central nervous system infections, osteoarticular and soft tissue infections and pneumonia. In immunocompromised patients, infections can involve any organ with a predilection for skin (as in our case), sinuses, lungs and central nervous system [4, 5, 7, 16].

The histological study and the microbiological culture are the main tools to reach a diagnosis. At the moment, molecular techniques are reserved for studies [17]. Histopathologic examination is helpful in determining the presence of an invasive fungal infection (e.g. positivity in the specific fungal stains, septate hyphae…), but it is not possible to establish the causative pathogen without culture because many molds have a similar appearance. Culture is also important for antifungal susceptibility testing [4, 5]. In a Spanish study analysing 60 isolates of *Scedosporium*, most of the strains (with exception of *S. prolificans*) were found susceptible for voriconazole and micafungin [18].

The prognosis is poor due to the comorbidities of patients and the multi-resistance of the *Scedosporium* species, with high MICs (Minimum Inhibitory Concentration) to most of antifungals tested. *S. apiospermum* shows in vitro susceptibility to voriconazole, posaconazole and in some cases to echinocandins [10]. Also, species and site of infection were shown to be statistically significant prognostic factors for overall global response [12]. Surgical resection or debridement is key in immunosuppressed patients. Its indication in the ESCMID and ECMM guidelines are the following: haemoptysis from a single cavitary lung lesion, progressive cavitary lung lesion, osteomyelitis or septic arthritis, resection of infected or colonized tissue before commencing immunosuppressive agents to prevent dissemination and infiltration into the pericardium great vessels, bone or thoracic sort tissue [4].

Overall mortality is 30% for immunocompetent patients and 44% for immunocompromised hosts [12]. The most at-risk patients are hematopoietic stem cell transplant recipients and the ones suffering from disseminated disease. Patients receiving voriconazole have longer survival time compared to patients treated with amphotericin B [4, 7, 12]. In a retrospective study of 107 voriconazole treated patients, the median therapy duration in treatment success cases was 180 days. Best success rate was seen in skin/subcutaneous tissues [7].

In conclusion, emerging fungal infections are increasing globally. These species are more difficult to diagnose and treat due to their resistance, which implies a higher mortality [19]. Therefore, clinicians must be more aggressive in the diagnosis, ruling out complications and treatment (including surgery if necessary) [4, 7]. More studies are needed to provide more information on all these aspects.

## Data Availability

All data generated or analysed during this study are included in this published article.
